# Curative Effect and Survival Assessment Comparing Gemcitabine and Cisplatin Versus Methotrexate, Vinblastine, Doxorubicin and Cisplatin as Neoadjuvant Therapy for Bladder Cancer: A Systematic Review and Meta-Analysis

**DOI:** 10.3389/fonc.2021.678896

**Published:** 2021-11-25

**Authors:** Tiange Wu, Yuqing Wu, Shuqiu Chen, Jianping Wu, Weidong Zhu, Hui Liu, Ming Chen, Bin Xu

**Affiliations:** ^1^ Surgical Research Center, Institute of Urology, Medical School of Southeast University, Nanjing, China; ^2^ Department of Urology, Zhongda Hospital Affiliated to Southeast University, Nanjing, China; ^3^ Department of Urology, Binhai County People’s Hospital, Yancheng, China; ^4^ Department of Urology, Zhongda Hospital Affiliated to Southeast University Lishui Branch, Nanjing, China

**Keywords:** bladder cancer, neoadjuvant chemotherapy, gemcitabine plus cisplatin, methotrexate plus vinblastine plus doxorubicin plus cisplatin, pathological response

## Abstract

**Background:**

Neoadjuvant chemotherapy has been accepted as an effective curative treatment for muscle-invasive bladder cancer patients and has resulted in better survival outcomes than radical cystectomy or a cisplatin-based regimen. In the present study, we aimed to compare the two most commonly used cisplatin-based neoadjuvant chemotherapies, gemcitabine plus cisplatin and methotrexate plus vinblastine plus doxorubicin plus cisplatin, by summarizing and analyzing clinical data and outcomes of published research.

**Methods:**

We searched for qualified studies that compared these two types of neoadjuvant chemotherapy, including 4 randomized controlled trials and 14 retrospective studies. Data and information on pathological responses and long-term survival studies were extracted and analyzed separately.

**Results:**

A total of 18 studies with 3116 patients were selected from 1188 studies, which contained data on pathological complete response, pathological partial response, and overall survival. In contrast to the results of previous studies, there was no significant difference in pathological complete response (odds ratio, 0.97; 95% confidence interval, 0.81-1.15), pathological partial response (odds ratio, 0.85; 95% confidence interval, 0.72-1.14), and overall survival (hazard ratio, 0.99; 95% confidence interval, 0.83-1.17) between GC and MVAC in this meta-analysis.

**Conclusion:**

No significant differences were observed between GC and MVAC in the muscle-invasive bladder cancer treatment due to the similar curative effect and parallel long survival outcomes due to the similar curative effect and parallel long survival outcomes. The priority selection of GC or MVAC in the clinic should be guided by further investigation, and the clinical standard strategy still counts on the results of more randomized controlled trials in the future.

## 1 Introduction

Bladder cancer is the second most common carcinoma in the urinary tract, and its occurrence rate continues to increase annually worldwide. Muscle-invasive bladder cancer (MIBC), an aggressive type of bladder cancer with a high risk of peripheral and distant metastasis, has been widely recognized as one of the primary causes of tumor-related death currently (1). Many randomized controlled trials (RCTs) have reported that cisplatin-based neoadjuvant chemotherapy (NCT) has superior curative effects to either radical cystectomy or locoregional treatment alone and could improve long-term survival outcomes. Recent reports also supported the ability of NCT to effectively decrease the morbidity and mortality of MIBC when combined with radical cystectomy, making NCT a clinically preferable option for treating MIBC (2).

However, the preferred regimen of cisplatin-based NCT for MIBC remains debatable. Considering the outcomes of the significant SWOG-8710 RCT, methotrexate, vinblastine, doxorubicin, and cisplatin (MVAC) were established as the most effective regimen in the NCT setting (3). In contrast, another RCT showed that gemcitabine plus cisplatin (GC) had an advantage in terms of survival outcomes, and therefore, this regimen has been increasingly applied in the NCT setting over MVAC (4).

In 2016, a two-step meta-analysis claimed that MVAC is superior in terms of overall survival of GC and might have the same treatment response rate as the latter, suggesting that MVAC should be the standard care in MIBC (5). On the contrary, another systematic review reporting comparative outcomes of the two chemotherapies two years later argued that GC ought to be used as the first-line chemotherapeutics during the treatment, as it showed better clinical efficacy than MVAC (6). However, results of both studies were not significant. In addition, the references cited in these studies might include repetitive samples. In the present study, we attempted to provide an independent and distinct conclusion with minimum possible bias. It could help to update the relevant information and offer clinical guidance for the future studies.

## 2 Materials and Methods

### 2.1 Documents Retrieval and Search Strategy

The standards of retrieved document were in line with the principle of participants, interventions, comparators, outcomes, and study design (PICOS),which were shown as follows: Participants, patients diagnosed as MIBC who were willing to undergo radical cystectomy and patients who underwent systemic neoadjuvant chemotherapy; Interventions, MIBC patients who underwent systemic NCT using MVAC or dense dose MVAC (ddMVAC); Comparators, MIBC patients who underwent systemic NCT using GC; Outcomes, comparison of pathologic (pathologic complete response (PCR), pathologic complete response (PPR) and prognostic outcomes [Overall Survival (OS)]; and Study design, both prospective RCTs and retrospective observational studies could be included for analyses.

We extensively searched PubMed, the Cochrane Central Search Library, and the Web of Science for related clinical trials and retrospective studies ranging from January 1, 2005, to September 30, 2021. The search terms used included bladder cancer, gemcitabine, cisplatin, methotrexate, vinblastine, and doxorubicin. The formula was as follows: (“GC” OR “gemcitabine and cisplatin/carboplatin” OR “gemcitabine/cisplatin” OR “gemcitabine plus cisplatin” OR “gemcitabine and cisplatin” OR “gemcitabine PLUS carboplatin” OR “gemcitabine/carboplatin”) AND (“MVAC” OR “Methotrexate, vinblastine, doxorubicin, and cisplatin” OR “Methotrexate/vinblastine/doxorubicin/cisplatin” OR “Methotrexate plus vinblastine plus doxorubicin plus cisplatin”) AND (“bladder cancer” OR “MIBC” OR “bladder tumor” OR “carcinoma” OR “tumor” OR “neoplasm”). The qualified studies were identified using the following inclusion criteria: A) Patients diagnosed with MIBC in accordance with the biopsy results. B) Patients voluntarily willing to undergo cisplatin-based neoadjuvant chemotherapy. C) Research included meaningful comparative results of GC versus MVAC, such as pathological complete response, pathological partial response, and overall survival outcomes. D) No overlapping samples represented in different studies.

### 2.2 Data Extraction

All data were extracted in the NoteExpress form and included the original title, data source, author, research objects, test and control measures, measurement and evaluation, statistical analysis, and conclusion derivation. We contacted the authors for the missing content wherever feasible. All objective data were extracted from the qualified publications by two investigators after an independent assessment. Controversial articles were re-evaluated by a third researcher. Odds ratios (ORs) were used to describe quantitative outcome indicators, including PCR and PPR. Hazard ratios (HRs) belonging to the survival analysis were used to evaluate OS. If the HRs were unavailable from the corresponding authors, we used Engauge Digitizer v10.8 to extract the Kaplan-Meier curves provided by articles to calculate HRs, lower confidence intervals, and upper confidence intervals (7).

### 2.3 Statistical Analysis

The meta-analysis was performed to summarize and analyze clinical outcomes from the literature, including PCR, PPR, and OS in both GC and MVAC groups. The Cochran Q test was applied to evaluate heterogeneity among studies with a significance level of *p*<0.05. When I^2 < 50% and *p*≥0.1, we performed the analysis using a fixed-effect model. If I^2≥50%, a random-effects model was used instead. Funnel plots were used to assess probable publication bias. All applicable data were analyzed with Review Manager 5.4 and Stata SE 12.0, to perform a meta-analysis. The analyses of PCR and PPR were performed with Review Manager version 5.4 (Cochrane, Oxford, U.K.), while the survival analysis was performed with Stata SE 12.0. All *p*-values were two-sided and set as *p <*0.05.

## 3 Results

### 3.1 Researches Screening and Risk Bias Assessment

**Figure 1:**
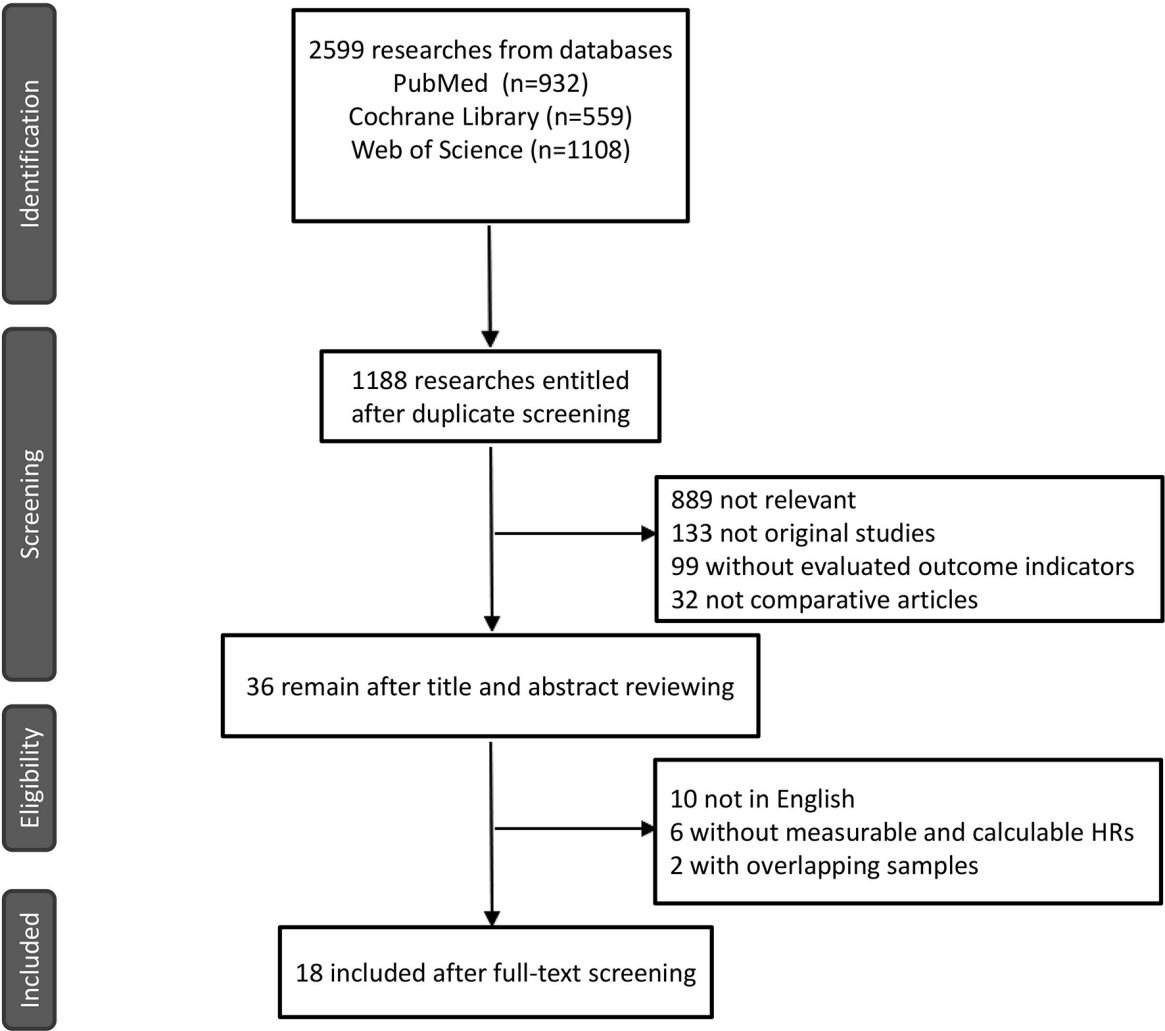
Flow chart of work process.

**Figure 2:**
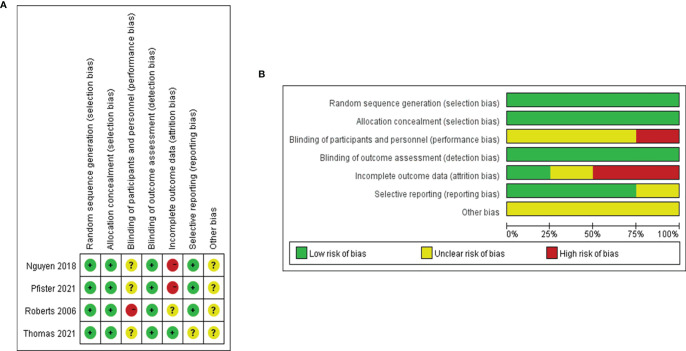
**(A)** Risk of bias graph based on Cochrane Risk-of-Bias Tool: review authors’ judgements about each risk of bias item presented as percentages across all included RCTs. **(B)** Risk of bias graph based on Cochrane Risk-of-Bias Tool: review authors’ judgements about each risk of bias item for each included RCT.

A total of 18 reports of 3116 patients receiving neoadjuvant chemotherapy were included, comprising 4 RCTs and 14 retrospective studies (8–25). All 18 articles provided comparative outcomes of PCR, in which seven studies had PPR outcomes and four studies conducted survival analyses. The risk of bias item presented in **Figure 1** showed a generally high quality of included articles, and the process of selecting qualified studies is displayed in **Figure 2**. Baseline characteristics are listed in **Table 1**. The quality of included articles was assessed by two reviewers with the use of Cochrane Risk-of-Bias Tool and Jadad for RCTs and Newcastle-Ottawa Scale (NOS) for case-control study. The items were separately shown in **Figures 2A, B** and **Appendix Tables 1, 2** (**Supplementary Material**).

**Table 1:** Baseline characteristics of included articles.

Author	Year	Country	Type	GC	MVAC	PCR	PPR	OS
Roberts (8)	2006	The USA	RCT	164	151	Y	N	Y
				NA	NA			
Dash (9)	2008	The USA	RCS	42	54	Y	Y	N
				4 cycle	4 cycle			
Weight (10)	2009	The USA	RCS	23	4	Y	N	N
				3 cycle	4 cycle			
Kaneko (11)	2011	Japan	RCS	22	9	Y	Y	N
				NA	NA			
Pal (12)	2012	The USA	RCS	24	22	Y	Y	N
				3-4 cycle	3-4 cycle			
Yeshchina (13)	2012	Japan	RCS	16	45	Y	Y	Y
				NA	NA			
Fairey (14)	2013	The USA	RCS	58	58	Y	Y	Y
				4 cycle	4 cycle			
Iwasaki (15)	2013	Japan	RCS	34	34	Y	N	N
				2-3 cycle	2-3 cycle			
Lee (16)	2013	The USA	RCS	41	31	Y	Y	N
				NA	NA			
Meijer (17)	2013	Netherland	RCS	45	117	Y	N	N
				2-4 cycle	2-4 cycle			
Zargar (18)	2015	The USA	RCS	602	183	Y	N	N
				3-4 cycle	3-4 cycle			
Galsky (19)	2015	The USA	RCS	146	66	Y	N	Y
				3-4 cycle	3-4 cycle			
Putte (20)	2016	The USA	RCS	115	51	Y	N	N
				4 cycle	4 cycle			
Nguyen (21)	2018	The USA	RCT	23	4	Y	Y	N
				3 cycle	3 cycle			
Peyton (22)	2018	The USA	RCS	204	46	Y	Y	Y
				3-4 cycle	3-4 cycle			
Pfister (23)	2021	France	RCT	198	199	Y	Y	N
				4 cycle	6 cycle			
Ruplin (24)	2020	The USA	RCS	76	33	Y	Y	N
				3-4 cycle	3-4 cycle			
Flaig (25)	2021	The USA	RCT	82	85	Y	Y	N
				4 cycle	4 cycle			

GC, Gemcitabine plus Cisplatin; MVAC, Methotrexate, vinblastine, doxorubicin, and cisplatin; PCR, Pathological Complete Response; PPR, Pathological Partial Response; OS, Overall Survival; RCT, Randomized controlled Trial; RCS, Retrospective Study; Y, Yes; N, No; NA, Not Available.

### 3.2 Treatment Response of GC vs. MVAC

#### 3.2.1 Pathological Complete Response (pT0)

**Figure 3:**
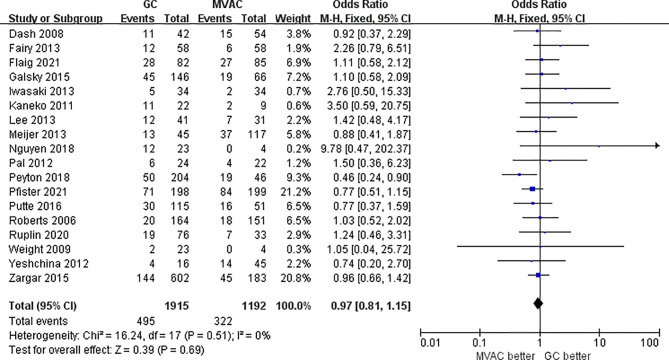
Forest plot of pathological complete response (PCR) to GC versus MVAC.

**Figure 4:**
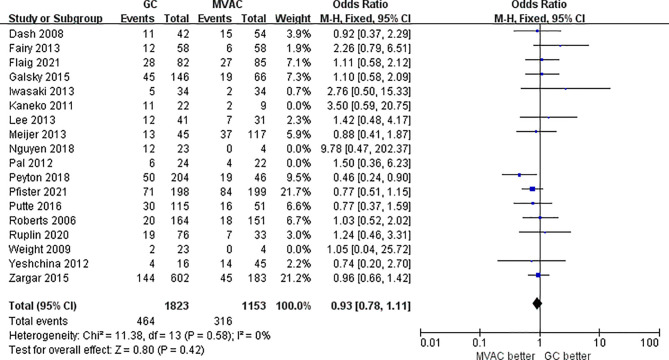
Forest plot of pathological complete response (PCR) to GC versus MVAC after removing studies with small sample sizes.

All 18 studies with 3116 patients compared the PCRs between GC and MVAC. The difference between GC and MVAC for PCR was insignificant (OR, 0.97; 95% confidence interval (CI), 0.81-1.15, *p*=0.69). No significant heterogeneity was found among the studies (*p*= 0.51, I^2 = 0%), and a fixed-effects model was used in the analysis (**Figure 3**). Four studies with relatively small sample sizes (< 50) were excluded for the sensitivity analysis to reduce possible bias. The analysis of the remaining 12 studies showed no significant difference (OR, 0.93; 95% CI, 0.78-1.11, *p*=0.42). No significant heterogeneity was found among the studies (*p*= 0.58, I^2 = 0%) (**Figure 4**).

#### 3.2.2 Pathological Partial Response (<T2)

**Figure 5:**
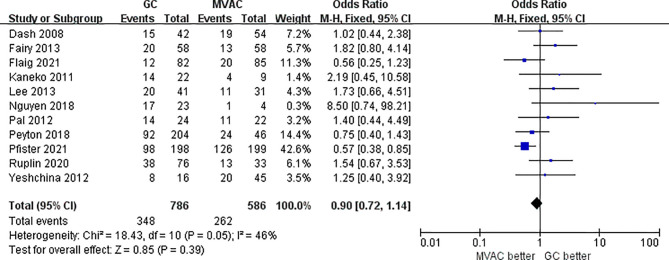
Forest plot of pathological partial response (PPR) to GC versus MVAC.

**Figure 6:**
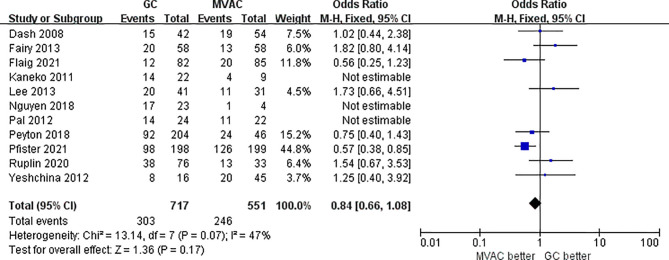
Forest plot of pathological partial response (PPR) to GC versus MVAC after removing studies with small sample sizes.

11 studies with 1372 patients reported the results of PPR to GC and MVAC regimens. The difference between GC and MVAC for PPR was insignificant (OR, 0.90; 95% CI, 0.72-1.14, *p*=0.39). No significant heterogeneity was found among the studies (*p*= 0.05, I^2 = 46%), and a fixed-effects model was used in the analysis (**Figure 5**). However, after removing 3 studies with small sample sizes under 50 to reduce inherent bias, the result generally appeared the same (OR, 0.84; 95% CI, 0.66-1.08, *p*=0.17). Significant heterogeneity was found among the studies (*p*= 0.07, I^2 = 47%) (**Figure 6**).

### 3.3 Overall Survival

**Figure 7:**
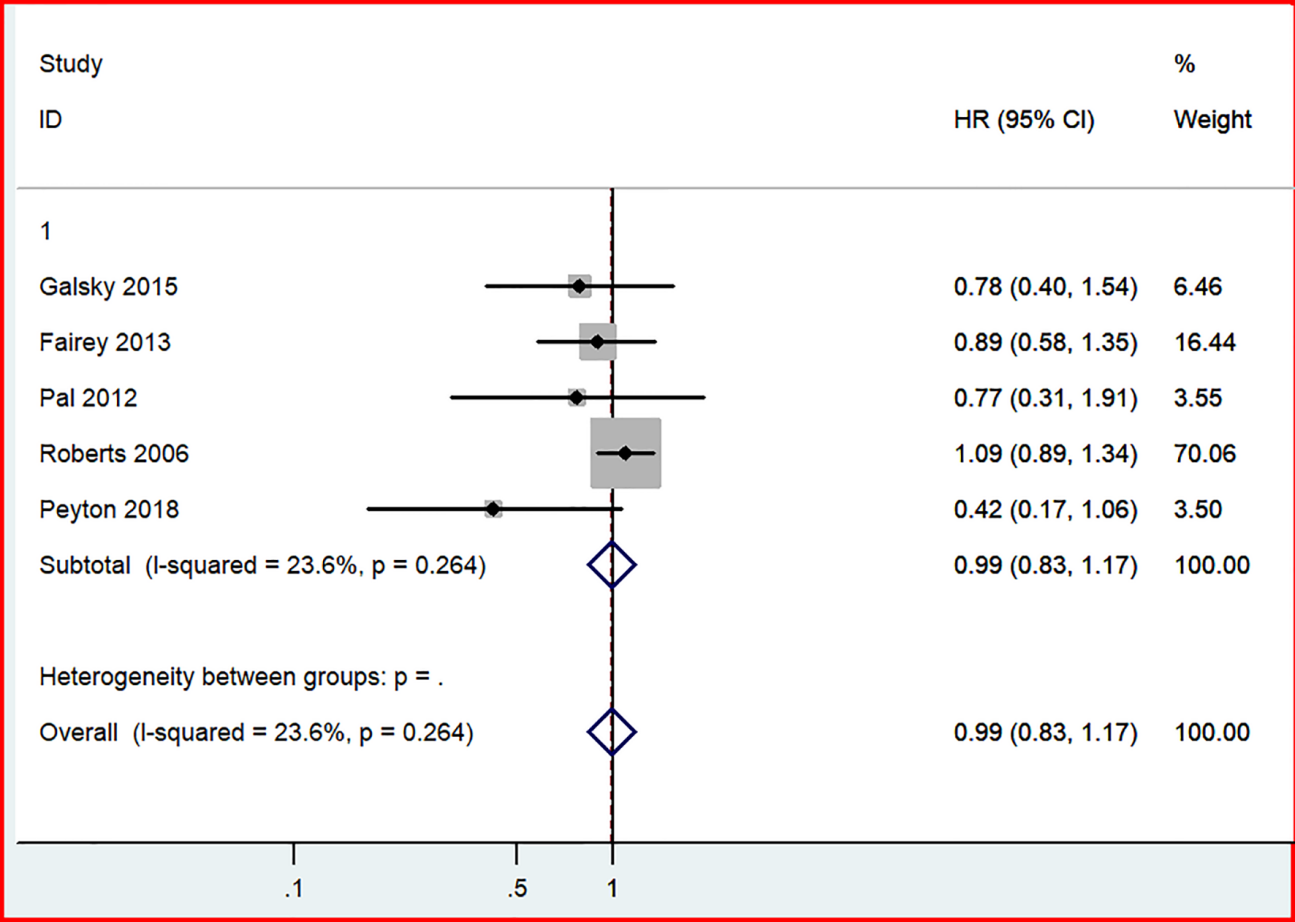
Forest plot of overall survival in comparison of GC versus MVAC.

In our analysis of long-term outcomes from four studies, including 689 patients, no significant difference in OS was discovered between the GC and MVAC groups (HR, 0.99; 95% CI, 0.83-1.17, *p*=0.868). No significant heterogeneity was not observed in this analysis (*p*= 0.264, I^2 = 23.6%) (**Figure 7**).

### 3.4 Publication Bias

**Figure 8:**
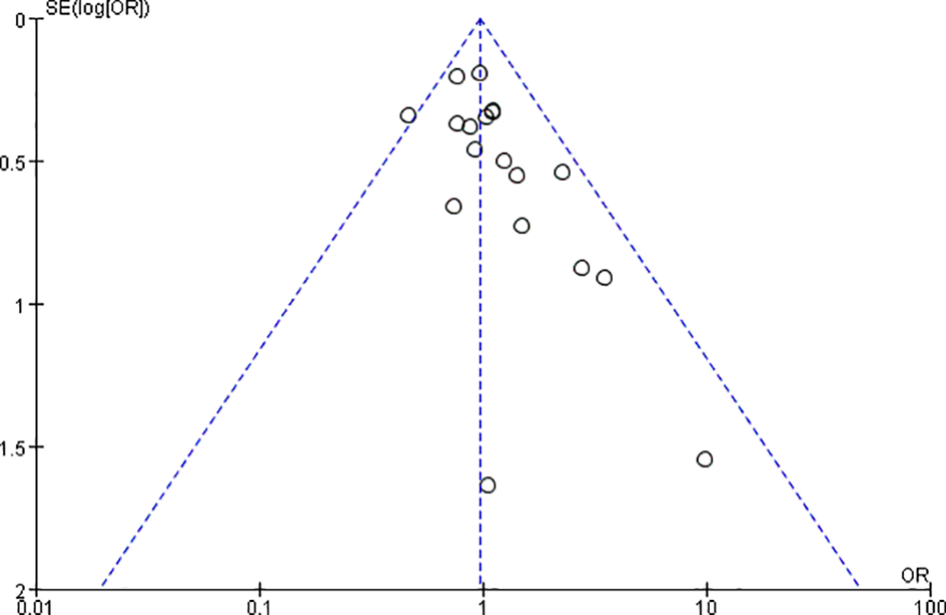
Funnel plot of PCR publication bias.

A funnel plot was established to evaluate publication bias among all 18 studies (**Figure 8**). Publication bias was not detected in the funnel plot or Egger’s test (data not shown).

## 4 Discussion

Since the 1980s, treatment of locally advanced and metastatic bladder cancer has been cisplatin-based (26). Several clinical trials have suggested MVAC as the standard therapy for almost 10 years (27). Although a survival advantage was seen with MVAC compared with cisplatin alone, MVAC still showed a high risk of nonnegligible toxicity. It was worth noting that ddMVAC showed better tolerability compared with classical dose of MVAC in previous researches, but there was no significant difference in OS outcome between the two regimens. Therefore, we did not treat them differently, since our analyses were partially focused on the prognosis of OS. The ddMVAC was more recommended in the regimen for the subgroup of patients pursuing persistent disease control, as was referred to in the review published by Vaibhav G Patel, et al. in 2020 (28). Thus, a less toxic but identical curative regimen is required. In 1995, GC was developed and found to be effective in many types of cancers, including bladder carcinoma. Since then it has been officially utilized for bladder cancer treatment as an alternative to MVAC (29, 30). However, clinical prioritization of individual regimen is still debatable.

According to the first quantitative analysis in 2016 comparing GC and MVAC as the two most commonly used chemotherapies in neoadjuvant settings, MVAC was suggested as the preferable regimen to cure MIBC with better long-term survival outcomes and similar clinical efficacy compared with GC (5). However, two years later, another meta-analysis claimed that GC was the standard guidance in MIBC treatment, considering its better curative effect than MVAC (6). Consequently, there is a dispute regarding which one of the two regimens could be acknowledged as the authoritative neoadjuvant chemotherapy for MIBC. Despite the similar purpose of these two meta-analyses, the different studies they included leaded to opposite conclusions. Under these circumstances, we believe it is necessary to add new studies and proceed with the research on this topic to obtain an updated conclusion precisely.

This study completed a comprehensive analysis, the conclusion of which was different from the previous analyses. In the present study, PCR and PPR were considered as indicators reflecting the clinical efficacy of GC and MVAC (PCR: 25.8% vs. 27.0%; PPR: 43.3% vs. 46.0%) among the 18 independent studies. Both types of pathological responses exhibited insignificant differences between the two groups of regimens, even when we removed studies with small samples for sensitivity analyses (PCR: 25.5% vs. 27.4%; PPR: 42.3% vs. 44.6%). These results further indicated the stability and reliability. The comparative result of PCR was more persuasive than that of PPR, in that the PCR analysis covered a larger number of patients who participated in RCTs. Despite the relatively better pathological responses to MVAC, a definite choice between the two therapies could still not be made because the difference was not statistically significant. As for the long-term survival outcome in 6 studies, no significant difference was observed in OS between the two groups of interest, though the MVAC also manifested a tendency of better prognosis. The HRs applied were directly extracted from the articles or identified from visualized Kaplan-Meier curves by Engauge Digitizer v10.8, which were more accurately calculated than other similar studies, which could be considered as an innovation.

What is more, it was debatable when it comes to the standard cycle number of treatments. 3 or 4 cycles have been recommended as the optimal manner for GC receiver, which could provide the adequate efficacy and avoid missing the best opportunity for radical cystectomy (31). However, disputes remained in choosing an appropriate treatment period of MVAC regimen. Although large-scale multicenter randomized trials were included in our study for quantitative synthesis and analyses, the numbers of MVAC cycles were different, ranging from 2 to 6 cycles. Among the studies included in our research, the PCR and PPR were respectively 5.9%-41.3% and 22.4%-62.2% for the numbers of cycles, which were less than or equal to 4, and they were respectively 42.2% and 63.3% for 6 two-week cycles. Consequently, 6 cycles of MVAC might have an edge over 2~4 cycles on pathological response for better operative opportunity but could lead to a postponed checkpoint that delays the surgery.

Some limitations could not be ignored in our updated research. First, double-blinding was difficult to achieve because of the nature of the intervention. Second, although we included 4 prospective RCTs, the sample size for the meta-analysis was still relatively small, given the main component was retrospective analyses pooled in our research, so the prospective data were still insufficient. Nevertheless, the retrospective studies were of generally high quality when rated with NOS. Moreover, the difference of effectiveness between GC and MVAC is vague, based on our updated conclusion. Therefore, though our research puts forward a different perspective from before, we were not able to propose instructive conclusions for regimen choice. When we looked up and attempted to explore the better option considering the side effects of toxicity, two small-scale RCTs (32, 33) comparing the drug toxicity were found, published in the beginning of 21st Century. However, in 2021, Pfister C. et al. performed the latest VESPER randomized phase III trial with a large sample size and prospectively demonstrated the superiority of ddMVAC (25), which was taken as strong evidence in our meta-analysis. The results of this large-scale clinical trial showed that although GC was more manageable with slighter asthenia and gastrointestinal side effects than ddMVAC, the latter had a higher local control rate and showed prominent advantage in pathological response. Due to this conflict, we anticipated more clinical trials concerning toxicity to provide rationales for medication in the future.

### 4.1 Conclusion

There is no significant difference between GC and MVAC, regarding the use of neoadjuvant chemotherapy to treat MIBC. Clinicians should develop treatment schemes counting on other factors, such as drug side effects, interaction with surgeries and treatment conditions. However, the conclusions drawn from our study should be interpreted cautiously. Large-scale and multicenter RCTs integrated with drug toxicity and cycle number comparison are demanded before final clinical guidance can be officially recommended as standards.

## Data Availability Statement

The original contributions presented in the study are included in the article/**Supplementary Material**. Further inquiries can be directed to the corresponding authors.

## Author Contributions

TW, MC, and HL designed the study. BX and YW conducted the study and maintained the data. SC, JW, and WZ analyzed the data and made the figures. All authors drafted and revised the paper. TW and YW reanalyzed the data and completed the final version of the manuscript and figures. All authors approved the final version of the manuscript.

## Funding

This study was funded by The National Natural Science Foundation of China (No. 81872089, 81370849, 81672551, 81300472, 81070592, 81202268, 81202034), Natural Science Foundation of Jiangsu Province (BK20161434, BL2013032, BK20150642 and BK2012336), Six talent peaks project in Jiangsu Province, Jiangsu Provincial Medical Innovation Team (CXTDA2017025), The National Key Research and Development Program of China (SQ2017YFSF090096), Jiangsu Provincial Key Research and Development Program(BE2019751), Innovative Team of Jiangsu Provincial (2017ZXKJQWO7), and Jiangsu Provincial Medical Talent (ZDRCA2016080).

## Conflict of Interest

The authors declare that the research was conducted in the absence of any commercial or financial relationships that could be construed as a potential conflict of interest.

## Publisher’s Note

All claims expressed in this article are solely those of the authors and do not necessarily represent those of their affiliated organizations, or those of the publisher, the editors and the reviewers. Any product that may be evaluated in this article, or claim that may be made by its manufacturer, is not guaranteed or endorsed by the publisher.
