# Supplementary material for: Curative Effect and Survival Assessment Comparing Gemcitabine and Cisplatin Versus Methotrexate, Vinblastine, Doxorubicin and Cisplatin as Neoadjuvant Therapy for Bladder Cancer: A Systematic Review and Meta-Analysis
Source: Front Oncol. 2021 Nov 25;11:678896. doi: 10.3389/fonc.2021.678896 (PMC8656312; doi:10.3389/fonc.2021.678896)
Supplement: Supplementary file 1 [file Table_1.docx]

**Appendix Table 1. Evaluation of the quality of literature in case control study using NOS**

| **Author** | **Year** | **Choice** | **Comparability** | **Exposure** | **Score** |
| --- | --- | --- | --- | --- | --- |
| Dash ^[9]^ | 2008 | 4 | 2 | 2 | 8 |
| Weight ^[10]^ | 2009 | 4 | 2 | 2 | 8 |
| Kaneko ^[11]^ | 2011 | 4 | 2 | 2 | 8 |
| Pal ^[12]^ | 2012 | 4 | 2 | 2 | 8 |
| Yeshchina ^[13]^ | 2012 | 4 | 2 | 2 | 8 |
| Fairey ^[14]^ | 2013 | 4 | 2 | 1 | 7 |
| Iwasaki ^[15]^ | 2013 | 4 | 2 | 2 | 8 |
| Lee ^[16]^ | 2013 | 4 | 2 | 2 | 8 |
| Meijer ^[17]^ | 2013 | 4 | 2 | 2 | 8 |
| Zargar ^[18]^ | 2015 | 4 | 2 | 1 | 7 |
| Galsky ^[19]^ | 2015 | 4 | 2 | 2 | 8 |
| Putte ^[20]^ | 2016 | 4 | 2 | 1 | 7 |
| Peyton ^[22]^ | 2018 | 4 | 2 | 2 | 8 |
| Ruplin ^[24]^ | 2020 | 4 | 2 | 2 | 8 |

**Appendix Table 2. Evaluation of the quality of literature in RCT study using Jadad**

| **Author** | **Year** | **Random sequence** | **Randomized hiding** | **Blind method** | **Withdrawal** | **Score** |
| --- | --- | --- | --- | --- | --- | --- |
| Roberts ^[8]^ | 2006 | 2 | 2 | 0 | 0 | 4 |
| Nguyen ^[21]^ | 2018 | 2 | 2 | 0 | 0 | 4 |
| Pfister ^[23]^ | 2021 | 2 | 2 | 0 | 1 | 5 |
| Flaig ^[25]^ | 2021 | 2 | 2 | 0 | 1 | 5 |
